# Poly[(μ_3_-pyridine-4-carboxyl­ato-κ^3^ *O*:*O*′:*N*)(pyridin-1-ium-4-carboxyl­ato-κ*O*)(thio­cyanato-κ*N*)cobalt(II)]

**DOI:** 10.1107/S1600536812044431

**Published:** 2012-11-03

**Authors:** Tristan Neumann, Julia Werner, Inke Jess, Christian Näther

**Affiliations:** a Institut für Anorganische Chemie, Christian-Albrechts-Universität Kiel, Max-Eyth-Strasse 2, 24118 Kiel, Germany

## Abstract

In the title compound, [Co(C_6_H_5_NO_2_)(NCS)(C_6_H_4_NO_2_)]_*n*_, the Co^2+^ cation is coordinated by one N and two O atoms of three bridging pyridine-4-carboxyl­ate anions, one O atom of one zwitterionic pyridinium-4-carboxyl­ate ligand and one terminal N-bonding thio­cyanate anion within a distorted N_2_O_3_ trigonal bipyramid. The bridging coordination mode of the ligands leads to the formation of layers parallel to (-101). N—H⋯O hydrogen-bonding inter­actions within the layers and S⋯S contacts of 3.257 (3) Å between the layers lead to the cohesion of the structure.

## Related literature

For general background information on the synthesis and properties of transition metal–thio­cyanate coordination polymers, see: Boeckmann & Näther (2010 ▶, 2011 ▶); Wöhlert *et al.* (2011 ▶).
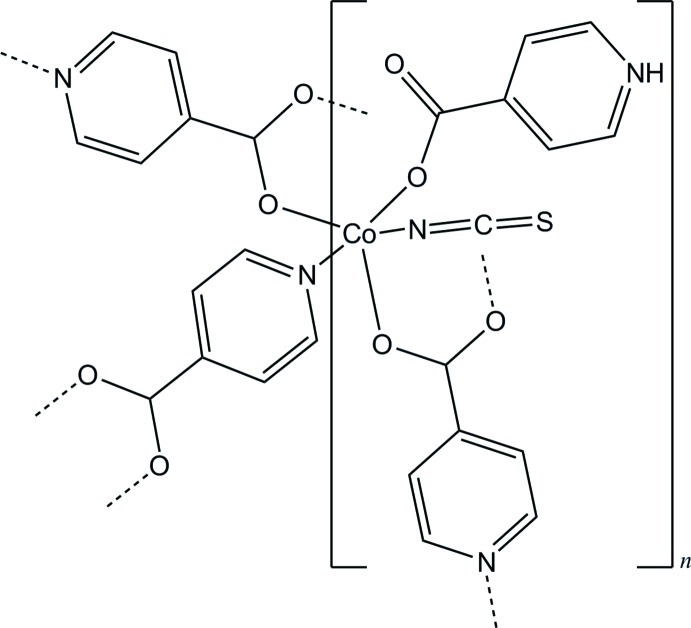


## Experimental

### 

#### Crystal data

[Co(C_6_H_5_NO_2_)(NCS)(C_6_H_4_NO_2_)]
*M* *_r_* = 362.22
Monoclinic, 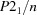

*a* = 8.7857 (7) Å
*b* = 13.5401 (8) Å
*c* = 12.2054 (9) Å
β = 95.740 (6)°
*V* = 1444.67 (18) Å^3^
*Z* = 4
Mo *K*α radiation
μ = 1.35 mm^−1^
*T* = 293 K
0.18 × 0.13 × 0.04 mm

#### Data collection

Stoe IPDS-2 diffractometer
Absorption correction: numerical (*X-SHAPE* and *X-RED32*; Stoe & Cie, 2008 ▶) *T*_min_ = 0.808, *T*_max_ = 0.954
12489 measured reflections
2844 independent reflections
2353 reflections with *I* > 2σ(*I*)
*R*_int_ = 0.041

#### Refinement

*R*[*F* ^2^ > 2σ(*F* ^2^)] = 0.045
*wR*(*F* ^2^) = 0.098
*S* = 1.13
2844 reflections
199 parameters
H-atom parameters constrained
Δρ_max_ = 0.46 e Å^−3^
Δρ_min_ = −0.39 e Å^−3^

### 

Data collection: *X-AREA* (Stoe & Cie, 2008 ▶); cell refinement: *X-AREA*; data reduction: *X-AREA*; program(s) used to solve structure: *SHELXS97* (Sheldrick, 2008 ▶); program(s) used to refine structure: *SHELXL97* (Sheldrick, 2008 ▶); molecular graphics: *XP* in *SHELXTL* (Sheldrick, 2008 ▶) and *DIAMOND* (Brandenburg, 2011 ▶); software used to prepare material for publication: *publCIF* (Westrip, 2010 ▶).

## Supplementary Material

Crystal structure: contains datablock(s) I, global. DOI: 10.1107/S1600536812044431/wm2684sup1.cif

Structure factors: contains datablock(s) I. DOI: 10.1107/S1600536812044431/wm2684Isup2.hkl

Additional supplementary materials:  crystallographic information; 3D view; checkCIF report

## Figures and Tables

**Table 1:** Selected bond lengths (Å)

Co1—O22	2.004 (2)
Co1—O21^i^	2.004 (2)
Co1—N1	2.010 (4)
Co1—O12	2.097 (2)
Co1—N21^ii^	2.146 (2)

Symmetry codes: (i) 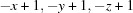; (ii) 
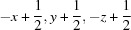
.

**Table 2:** Hydrogen-bond geometry (Å, °)

*D*—H⋯*A*	*D*—H	H⋯*A*	*D*⋯*A*	*D*—H⋯*A*
N11—H1*N*⋯O11^iii^	0.86	1.80	2.561 (4)	147

Symmetry code: (iii) 
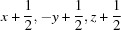
.

**Fig. 1.**
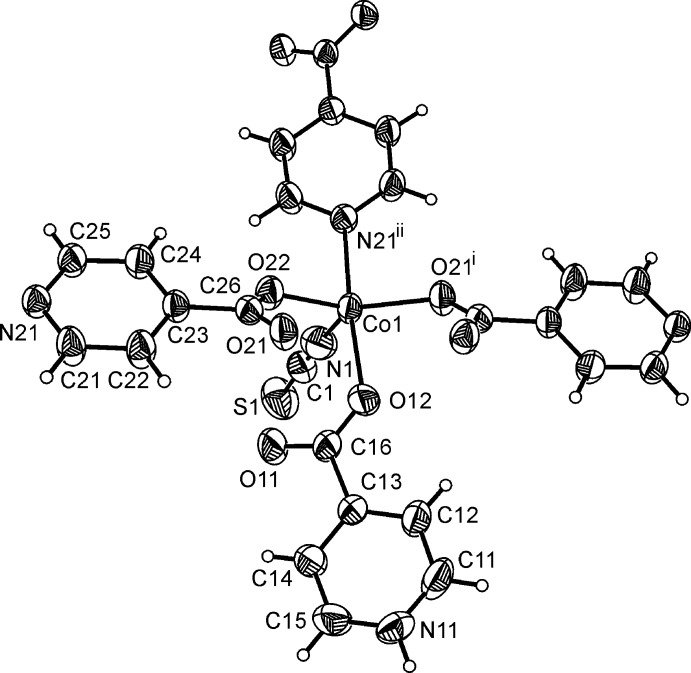
: The coordination environment of the Co2+ cation in the title compound with labelling and displacement ellipsoids drawn at the 50% probability level. [Symmetry codes: i = -x+1,-y+1,-z+1, ii = -x+1/2, y+1/2, -z+1/2.]

**Fig. 2.**
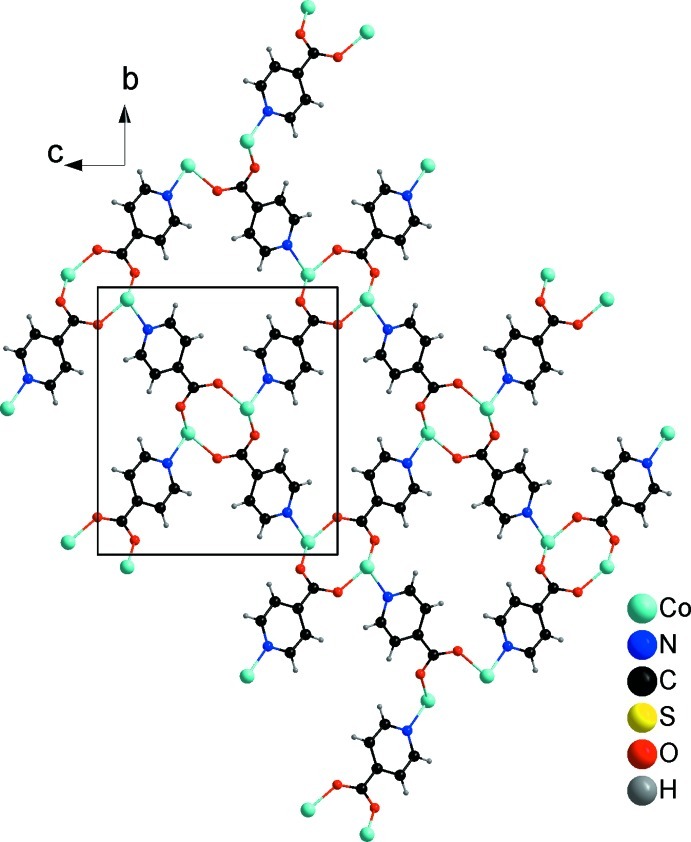
: The layers parallel to (101) in the title compound in a projection along the a axis. For clarity, the non-bridging ligands and the thiocyanato anions are not shown.

**Fig. 3.**
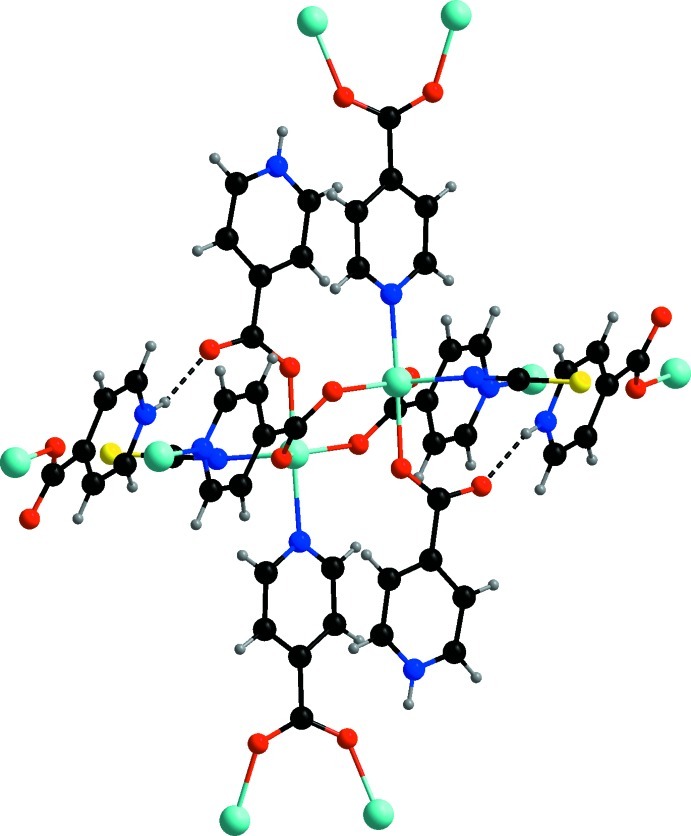
: The N—H···O hydrogen bonding interactions (dashed lines) within the layers in the crystal structure of the title compound.

## supplementary crystallographic information

### Comment

The title compound was prepared within a project on the synthesis and properties of transition metal thiocyanato coordination polymers (Boeckmann & Näther, 2010, 2011; Wöhlert *et al.*, 2011). During our attempts to prepare a one-dimensional coordination polymer based on pyridine-4-carboxylic acid as a co-ligand, crystals of the title compound, [Co(NCS)(C_6_H_4_NO_2_)(C_6_H_5_NO_2_)], (I), were obtained serendipitously and characterized by single crystal X-ray diffraction.

In the crystal structure of (I), the cobalt(II) cation is coordinated by one terminally *O*-bonded pyridinium-4-carboxylate ligand, one terminally *N*-bonded thiocyanate anion, one *N*-bonded *µ*-1,3,6-bridging pyridine-4-carboxylate and two *O*-bonded *µ*-1,3,6-bridging pyridine-4-carboxylate anions (Fig. 1). The coordination polyhedron of the Co^2+^ cations can be described as a distorted trigonal bipyramid (Fig. 1, Table 1).

The Co^2+^ cations are *µ*-1,3 bridged *via* pyridine-4-carboxylato anions into dimers, which are further connected into layers parallel to (101) (Fig. 2). The Co···Co distance within the dimer amounts to 3.4951 (6) Å. Within the layers N—H···O hydrogen bonding between the bridging pyridine-4-carboxylate anions and the non-bridging pyridinium carboxylate ligands (Fig. 3 and Table 2) is present. A short S···S contact of 3.257 (3) Å between the layers is also observed.

### Experimental

Cobalt thiocyanate and pyridine-4-carboxylic acid were purchased from Alfa Aesar. The title compound was prepared by the reaction of 43.8 mg Co(NCS)_2_ (0.25 mmol), and 61.6 mg pyridine-4-carboxylic acid (0.50 mmol) in 1.5 mL ethanol at 354 K in a closed 10 ml glas culture tube. After several days pink block-shaped crystals of the title compound were obtained.

### Refinement

The C-H and N-H H atoms were localized in a difference map but were positioned with idealized geometry and were refined isotropically with *U*_iso_(H) = 1.2 *U*_eq_(C,N) using a riding model with C—H = 0.93 Å and N—H = 0.86 Å.

### Crystal data

**Table:**

[Co(C_6_H_5_NO_2_)(NCS)(C_6_H_4_NO_2_)]	*F*(000) = 732
*M**_r_* = 362.22	*D*_x_ = 1.665 Mg m^−^^3^
Monoclinic, *P*2_1_/*n*	Mo *K*α radiation, λ = 0.71073 Å
Hall symbol: -P 2yn	Cell parameters from 12489 reflections
*a* = 8.7857 (7) Å	θ = 2.3–26.0°
*b* = 13.5401 (8) Å	µ = 1.35 mm^−^^1^
*c* = 12.2054 (9) Å	*T* = 293 K
β = 95.740 (6)°	Block, pink
*V* = 1444.67 (18) Å^3^	0.18 × 0.13 × 0.04 mm
*Z* = 4	

### Data collection

**Table:**

Stoe IPDS-2 diffractometer	2844 independent reflections
Radiation source: fine-focus sealed tube	2353 reflections with *I* > 2σ(*I*)
Graphite monochromator	*R*_int_ = 0.041
ω scans	θ_max_ = 26.0°, θ_min_ = 2.3°
Absorption correction: numerical (*X-SHAPE* and *X-RED32*; Stoe & Cie, 2008)	*h* = −9→10
*T*_min_ = 0.808, *T*_max_ = 0.954	*k* = −16→16
12489 measured reflections	*l* = −15→15

### Refinement

**Table:**

Refinement on *F*^2^	Primary atom site location: structure-invariant direct methods
Least-squares matrix: full	Secondary atom site location: difference Fourier map
*R*[*F*^2^ > 2σ(*F*^2^)] = 0.045	Hydrogen site location: inferred from neighbouring sites
*wR*(*F*^2^) = 0.098	H-atom parameters constrained
*S* = 1.13	*w* = 1/[σ^2^(*F*_o_^2^) + (0.0434*P*)^2^ + 0.689*P*] where *P* = (*F*_o_^2^ + 2*F*_c_^2^)/3
2844 reflections	(Δ/σ)_max_ < 0.001
199 parameters	Δρ_max_ = 0.46 e Å^−^^3^
0 restraints	Δρ_min_ = −0.39 e Å^−^^3^

### Special details

**Table:**

Geometry. All esds (except the esd in the dihedral angle between two l.s. planes) are estimated using the full covariance matrix. The cell esds are taken into account individually in the estimation of esds in distances, angles and torsion angles; correlations between esds in cell parameters are only used when they are defined by crystal symmetry. An approximate (isotropic) treatment of cell esds is used for estimating esds involving l.s. planes.
Refinement. Refinement of F^2^ against ALL reflections. The weighted R-factor wR and goodness of fit S are based on F^2^, conventional R-factors R are based on F, with F set to zero for negative F^2^. The threshold expression of F^2^ > 2sigma(F^2^) is used only for calculating R-factors(gt) etc. and is not relevant to the choice of reflections for refinement. R-factors based on F^2^ are statistically about twice as large as those based on F, and R- factors based on ALL data will be even larger.

### Fractional atomic coordinates and isotropic or equivalent isotropic displacement parameters (Å^2^)

**Table:**

	*x*	*y*	*z*	*U*_iso_*/*U*_eq_	
Co1	0.55767 (5)	0.54579 (3)	0.37683 (3)	0.04212 (15)	
N1	0.6768 (4)	0.5335 (2)	0.2453 (3)	0.0650 (8)	
C1	0.7529 (4)	0.5189 (3)	0.1751 (3)	0.0569 (9)	
S1	0.85857 (16)	0.50015 (10)	0.07668 (11)	0.0870 (4)	
O11	0.7180 (4)	0.3217 (2)	0.3482 (2)	0.0961 (12)	
N11	1.0561 (4)	0.2118 (2)	0.6656 (2)	0.0602 (8)	
H1N	1.1155	0.1797	0.7133	0.072*	
C11	1.0285 (5)	0.3058 (3)	0.6833 (3)	0.0691 (11)	
H11	1.0737	0.3363	0.7467	0.083*	
O12	0.7060 (4)	0.44642 (18)	0.4654 (2)	0.0746 (9)	
C12	0.9336 (5)	0.3592 (3)	0.6092 (3)	0.0626 (10)	
H12	0.9141	0.4255	0.6219	0.075*	
C13	0.8682 (4)	0.3134 (2)	0.5164 (3)	0.0472 (8)	
C14	0.9021 (6)	0.2159 (3)	0.4995 (3)	0.0731 (13)	
H14	0.8619	0.1840	0.4356	0.088*	
C15	0.9951 (6)	0.1659 (3)	0.5768 (3)	0.0797 (14)	
H15	1.0155	0.0993	0.5667	0.096*	
C16	0.7540 (5)	0.3656 (3)	0.4352 (3)	0.0585 (10)	
N21	0.0790 (3)	0.15794 (17)	0.20950 (19)	0.0409 (6)	
C21	0.1656 (4)	0.1340 (2)	0.3023 (3)	0.0541 (9)	
H21	0.1687	0.0682	0.3243	0.065*	
O21	0.3758 (3)	0.35997 (15)	0.50108 (16)	0.0480 (5)	
O22	0.3902 (3)	0.44629 (15)	0.34512 (17)	0.0468 (5)	
C22	0.2501 (4)	0.2017 (2)	0.3666 (3)	0.0525 (9)	
H22	0.3069	0.1817	0.4311	0.063*	
C23	0.2502 (4)	0.2995 (2)	0.3350 (2)	0.0392 (7)	
C24	0.1599 (4)	0.3245 (2)	0.2392 (3)	0.0520 (8)	
H24	0.1560	0.3896	0.2146	0.062*	
C25	0.0765 (4)	0.2528 (2)	0.1808 (3)	0.0503 (8)	
H25	0.0149	0.2714	0.1177	0.060*	
C26	0.3462 (4)	0.3749 (2)	0.3992 (2)	0.0397 (7)	

### Atomic displacement parameters (Å^2^)

**Table:**

	*U*^11^	*U*^22^	*U*^33^	*U*^12^	*U*^13^	*U*^23^
Co1	0.0532 (3)	0.0318 (2)	0.0380 (2)	−0.00216 (19)	−0.01220 (16)	−0.00046 (16)
N1	0.061 (2)	0.0587 (18)	0.076 (2)	0.0046 (16)	0.0107 (17)	−0.0018 (16)
C1	0.055 (2)	0.0454 (18)	0.070 (2)	−0.0019 (16)	0.0022 (19)	−0.0096 (16)
S1	0.0904 (9)	0.0886 (8)	0.0864 (8)	−0.0032 (7)	0.0312 (7)	−0.0235 (7)
O11	0.144 (3)	0.0572 (16)	0.0716 (18)	0.0320 (18)	−0.066 (2)	−0.0231 (14)
N11	0.0593 (19)	0.0640 (19)	0.0532 (17)	0.0088 (15)	−0.0147 (14)	0.0118 (14)
C11	0.078 (3)	0.066 (2)	0.056 (2)	−0.016 (2)	−0.031 (2)	0.0079 (18)
O12	0.100 (2)	0.0499 (14)	0.0645 (15)	0.0289 (14)	−0.0373 (15)	−0.0134 (12)
C12	0.077 (3)	0.0465 (18)	0.058 (2)	−0.0028 (18)	−0.0265 (19)	−0.0022 (15)
C13	0.0520 (19)	0.0437 (16)	0.0430 (16)	0.0054 (14)	−0.0095 (14)	−0.0023 (13)
C14	0.105 (3)	0.057 (2)	0.049 (2)	0.030 (2)	−0.031 (2)	−0.0147 (17)
C15	0.109 (4)	0.063 (2)	0.061 (2)	0.037 (2)	−0.023 (2)	−0.0084 (19)
C16	0.072 (2)	0.0440 (18)	0.0532 (19)	0.0084 (17)	−0.0277 (17)	−0.0045 (15)
N21	0.0492 (15)	0.0369 (13)	0.0350 (12)	−0.0022 (11)	−0.0040 (11)	−0.0004 (10)
C21	0.080 (2)	0.0332 (15)	0.0446 (17)	−0.0082 (15)	−0.0168 (16)	0.0043 (13)
O21	0.0702 (15)	0.0338 (10)	0.0367 (11)	−0.0032 (10)	−0.0108 (10)	−0.0008 (8)
O22	0.0565 (13)	0.0368 (11)	0.0444 (11)	−0.0065 (10)	−0.0086 (10)	0.0029 (9)
C22	0.076 (2)	0.0396 (16)	0.0373 (15)	−0.0055 (16)	−0.0168 (15)	0.0026 (12)
C23	0.0497 (18)	0.0340 (14)	0.0324 (14)	−0.0022 (13)	−0.0037 (13)	−0.0027 (11)
C24	0.066 (2)	0.0332 (15)	0.0518 (18)	−0.0011 (14)	−0.0194 (16)	0.0025 (13)
C25	0.063 (2)	0.0376 (16)	0.0458 (17)	0.0009 (15)	−0.0183 (15)	−0.0002 (13)
C26	0.0462 (17)	0.0309 (14)	0.0402 (15)	0.0021 (12)	−0.0054 (13)	−0.0014 (11)

### Geometric parameters (Å, º)

**Table:**

Co1—O22	2.004 (2)	C14—C15	1.364 (5)
Co1—O21^i^	2.004 (2)	C14—H14	0.9300
Co1—N1	2.010 (4)	C15—H15	0.9300
Co1—O12	2.097 (2)	N21—C25	1.331 (4)
Co1—N21^ii^	2.146 (2)	N21—C21	1.339 (4)
N1—C1	1.155 (5)	N21—Co1^iii^	2.146 (2)
C1—S1	1.610 (4)	C21—C22	1.376 (4)
O11—C16	1.230 (4)	C21—H21	0.9300
N11—C15	1.316 (5)	O21—C26	1.261 (3)
N11—C11	1.318 (5)	O21—Co1^i^	2.004 (2)
N11—H1N	0.8600	O22—C26	1.253 (3)
C11—C12	1.373 (5)	C22—C23	1.379 (4)
C11—H11	0.9300	C22—H22	0.9300
O12—C16	1.242 (4)	C23—C24	1.388 (4)
C12—C13	1.366 (4)	C23—C26	1.495 (4)
C12—H12	0.9300	C24—C25	1.373 (4)
C13—C14	1.374 (5)	C24—H24	0.9300
C13—C16	1.514 (4)	C25—H25	0.9300
			
O22—Co1—O21^i^	136.53 (10)	N11—C15—H15	119.9
O22—Co1—N1	102.78 (12)	C14—C15—H15	119.9
O21^i^—Co1—N1	120.67 (12)	O11—C16—O12	128.1 (3)
O22—Co1—O12	94.28 (10)	O11—C16—C13	115.8 (3)
O21^i^—Co1—O12	84.54 (9)	O12—C16—C13	116.0 (3)
N1—Co1—O12	90.72 (13)	C25—N21—C21	116.7 (3)
O22—Co1—N21^ii^	90.97 (9)	C25—N21—Co1^iii^	124.0 (2)
O21^i^—Co1—N21^ii^	91.26 (9)	C21—N21—Co1^iii^	119.1 (2)
N1—Co1—N21^ii^	88.66 (12)	N21—C21—C22	123.3 (3)
O12—Co1—N21^ii^	174.71 (11)	N21—C21—H21	118.3
C1—N1—Co1	173.2 (3)	C22—C21—H21	118.3
N1—C1—S1	179.2 (4)	C26—O21—Co1^i^	130.33 (18)
C15—N11—C11	121.6 (3)	C26—O22—Co1	132.53 (19)
C15—N11—H1N	119.2	C21—C22—C23	119.7 (3)
C11—N11—H1N	119.2	C21—C22—H22	120.1
N11—C11—C12	120.6 (3)	C23—C22—H22	120.1
N11—C11—H11	119.7	C22—C23—C24	117.0 (3)
C12—C11—H11	119.7	C22—C23—C26	121.7 (3)
C16—O12—Co1	128.6 (2)	C24—C23—C26	121.4 (3)
C13—C12—C11	119.0 (3)	C25—C24—C23	119.7 (3)
C13—C12—H12	120.5	C25—C24—H24	120.2
C11—C12—H12	120.5	C23—C24—H24	120.2
C12—C13—C14	118.7 (3)	N21—C25—C24	123.5 (3)
C12—C13—C16	121.8 (3)	N21—C25—H25	118.2
C14—C13—C16	119.4 (3)	C24—C25—H25	118.2
C15—C14—C13	119.8 (3)	O22—C26—O21	126.8 (3)
C15—C14—H14	120.1	O22—C26—C23	116.0 (2)
C13—C14—H14	120.1	O21—C26—C23	117.2 (3)
N11—C15—C14	120.1 (4)		

Symmetry codes: (i) −*x*+1, −*y*+1, −*z*+1; (ii) −*x*+1/2, *y*+1/2, −*z*+1/2; (iii) −*x*+1/2, *y*−1/2, −*z*+1/2.

### Hydrogen-bond geometry (Å, º)

**Table:**

*D*—H···*A*	*D*—H	H···*A*	*D*···*A*	*D*—H···*A*
N11—H1*N*···O11^iv^	0.86	1.80	2.561 (4)	147

Symmetry code: (iv) *x*+1/2, −*y*+1/2, *z*+1/2.
